# A Retrospective Study of a Chinese Traditional Medicine YIKEER in the Treatment of Verruca Patients in Liaoning District

**DOI:** 10.1155/2019/9896148

**Published:** 2019-12-31

**Authors:** S. B. Jiang, Y. S. Lu, Y. H. Zhang, Y. Wu, H. X. Wang, X. H. Gao, H. D. Chen

**Affiliations:** Department of Dermatology, The First Hospital of China Medical University, Shenyang, China

## Abstract

**Background:**

There are many possible ways to treat verruca, but no one is the single perfect treatment. YIKEER is a kind of compound preparation of Chinese traditional medicine, which has been used in the treatment of verruca for several years.

**Aim:**

To confirm the effects of YIKEER for verruca.

**Method:**

Patients with verruca vulgaris, verruca plantaris, or verruca plana were instructed to apply YIKEER stock solution or diluent to the lesions once or twice daily for 5–7 days. Then, the YIKEER was ceased for 3–4 days, and sea buckthorn oil was used for wound repairing. The total procession was defined as one session.

**Result:**

Respective 88.05% verruca vulgaris patients, 86.03% verruca plantaris patients, and 82.42% verruca plana patients achieved complete response. Most patients gained complete or partial responses after 4 treatment sessions. The percentage of patients who achieved at least 50% improvement was 90.34% for verruca vulgaris, 90.60% for verruca plantaris, and 80.91% for verruca plana after 4-session treatment. The efficacy of verruca vulgaris or verruca plantaris was better than that of verruca plana.

**Conclusion:**

YIKEER is an effective, safe, and well-tolerated agent for treating verruca including verruca vulgaris, verruca plantaris, and verruca plana.

## 1. Introduction

Verruca can be divided into four typical types, including verruca vulgaris, verruca plantaris, verruca plana, and genital warts. They are caused by different strains of human papilloma virus (HPV) and present as benign epidermal proliferation. Verruca vulgaris is known as a common wart, and it tends to affect epithelial tissues and mucous membranes [1]. Verruca plantaris tends to occur at points on the foot where the most pressure is applied. The virus is thought to make its way into the foot via small abrasions in the stratum corneum [2]. Verruca plana is represented as a slightly elevated, flat-topped papule. It is relatively common in teenagers and does not always show Koebner's phenomenon which is a very useful clue for the clinical diagnosis [3].

There are many possible ways to treat verruca including topical medications, intralesional immunotherapy, cryotherapy, laser, and photodynamic therapy [4]. These treatments are considered to be similar in terms of efficacy [5]. The choice may be determined by factors such as wart location, availability of treatments at individual centers, convenience of use, aesthetic impact, patient preference, cost, and adverse effects. Besides, treatment responses are various with different types of verruca. Verruca plantaris tends to be more resistant to treatment than verruca at other sites. Warts with short-term duration are more likely to be cleared than those with long-term duration [5]. Although many local treatments are available, the problems in improving efficacy, decreasing recurrence rate, and ulcer formation still need to be solved. In some cases, verruca continues to increase in size and distribution, and it may become more resistant to treatment over time [6–8].

Natural medicines, including traditional Chinese medicines (TCMs), are commonly used for the treatment of skin disorders, especially for topical use. For example, Chyawanprash shows a protective effect on skin photoaging by inhibiting roughness, erythema, and edema in UVB-induced hairless mice [9]; extracts and bioactive compounds from *Hygrophila auriculata* have been found to possess antimicrobial, antioxidant, and anti-inflammatory activities and can alleviate skin itching and edema [10]; juglone and its synthetic triazolyl analogues present cytotoxic activity against skin cancer [11].

YIKEER is a kind of compound preparation of TCM. The main ingredients include *Brucea javanica*, *Folium Isatidis*, Radix sophorae flavescentis, *Lonicera japonica*, *Astragalus membranaceus*, *Fructus cnidii*, *Galla chinensis* [12, 13]. Phytochemical studies revealed that *Brucea javanica* is a rich source of quassinoids and oil-like lipids [14] and can make the epithelial cell degeneration and necrosis. Folium isatidis and Radix sophorae flavescentis have antibacterial, antiviral, antipyretic, and anti-inflammatory properties and can promote immunological response [15]. *Lonicera japonica*, *Astragalus membranaceus*, *Fructus cnidii*, and *Galla chinensis* have been reported to have many biological activities, such as antioxidative, an-inflammatory, antibacterial, antiviral, and antifungal activities [16–18].

In China, YIKEER has been used in the treatment of verruca for several years [12, 13, 19]. The present retrospective study summarized the clinical results of YIKEER on verruca patients in Liaoning district.

## 2. Method

### 2.1. Enrollment of Patients

The study was conducted in 5 cities of Liaoning district, including Shenyang, Huludao, Dandong, Fuxin, and Jinzhou. The period of study was from January 2014 to October 2018. The patients were diagnosed as verruca vulgaris, verruca plantaris, or verruca plana, according to the diagnostic criteria [20]. The exclusion criteria of patients were pregnancy or lactating at the time of admission, allergic to any of the components of the study medications, with allergic skin diseases such as eczema, contact dermatitis, and other serious skin diseases, with other infectious diseases such as human immunodeficiency virus (HIV) infection, hepatitis B or C virus infection, or syphilis.

#### 2.2. Components of the Medicine

The YIKEER kit was manufactured by Beijing PaiteBoen Biological Technology Co., Ltd. The components of the YIKEER liquid contained 5 main ingredients including *Folium isatidis* (1840 mg/L), Radix sophorae flavescentis (1600 mg/L), *Lonicera japonica* (1027 mg/L), *Fructus kochiae* (208 mg/L), and gallnut (5568 mg/L) and 4 secondary ingredients including *Astragalus membranaceus, Brucea javanica, Galla Chinensis,* and *Fructus cnidii.* A bottle of sea buckthorn oil was also included in the kit for repairing the wound.

### 2.3. Treatment Method

Patients with verruca vulgaris or verruca plantaris were instructed to apply YIKEER to the lesions with plastic occluding for at least 2 hours once daily for 5–6 days. For hard and big verruca plana, YIKEER was applied twice daily for 7 days without occlusion. For short-term and multiple verruca plana, the YIKEER was diluted to 1 : 10 to 1 : 20 with water and compressed with gauze for 15 to 20 minutes twice daily for 5–6 days. Then, the YIKEER was ceased for 3–4 days, and sea buckthorn oil was used. The total procession was defined as one session. If the warts did not disappear, another session was required. The maximal treatment period was 3 months (9 sessions). If the warts disappeared, the 1 : 20 diluted YIKEER was applied to the local skin for 2 weeks to maintain the efficacy and prevent the recurrence.

During the treatment period, if any allergic or aggravated signs including itching, severe erythema, severe edema, or exudation appeared, the drug should be stopped immediately and sea buckthorn oil was applied until the conditions improved.

### 2.4. Efficacy Assessments

The patients were required to take photos at baseline and when each session completed. Two investigators separately evaluated the clinical response based on the photos. The efficacy of the treatment was assessed by counting the number of lesions as follows: complete response: disappearance of all lesions; partial response (3 grades): excellent (75% < 100%), good (50% < 75%), poor (25% < 50%); no response: <25% reduction in number. The recurrence was assessed by inquiring the patients by telephone at 3-month follow-up.

### 2.5. Statistical Analysis

SPSS Version 20.0 (SPSS, Inc., Chicago, IL, USA) was used for all analyses. Nonparametric tests including Kruskal–Wallis test and Mann–Whitney test were used for comparisons. A *p*-value of <0.05 was considered to indicate statistical significance.

## 3. Results

### 3.1. Patient Characteristics

A total of 1860 patients with 614 verruca vulgaris patients (51.79% male and 48.21% female), 716 verruca plantaris patients (47.49% male and 52.51% female), and 530 verruca plana patients (31.70% male and 68.30% female) were recruited in the study from January 2014 to October 2018. The mean age of patients with verruca vulgaris, verruca plantaris, and verruca plana was 36 years, 33 years, and 29 years, respectively. During the period, respective 28 verruca vulgaris, 28 verruca plantaris, and 18 verruca plana patients did not return for the follow-up visit. So, a total of 1786 patients, including 586 verruca vulgaris, 688 verruca plantaris, and 512 verruca plana patients, completed the treatment and evaluation. The demographic data are summarized in Table 1.

### 3.2. The Overall Efficacies

For verruca vulgaris, verruca plantaris, or verruca plana, respective 88.05%, 86.03%, and 82.42% patients achieved complete response, and only 4.78%, 4.37%, and 7.42% patients showed no response to the overall treatments (Tables 2–4). No significant differences were observed among the efficacies of the three diseases (*χ*^2^ = 4.311, *p* > 0.05). The treatment duration of verruca vulgaris or verruca plantaris was at least 2 sessions and that of verruca plana was at least 3 sessions, and most patients needed 4 or even more sessions (Table 1). For the three diseases, most patients gained complete or partial responses after 4 treatment sessions, and the number of patients gradually decreased with the continuous treatment sessions.

**Figure 1:**
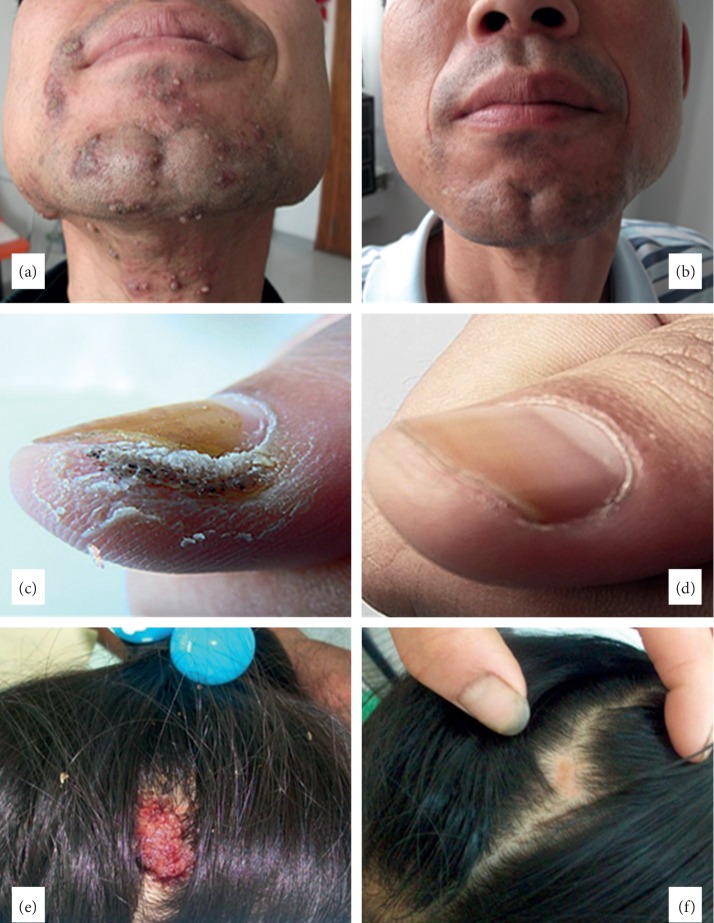
Three patients with verruca vulgaris before (a, c, e) and after (b, d, f) the treatment.

After 1 session of treatment, about 7% patients achieved at least 50% improvement of verruca vulgaris. The percentage markedly increased to more than 50% after 2 sessions of treatment and to more than 67% after 3 sessions of treatment. It reached a peak percentage of 90.34% after 4 sessions of treatment. After that, the percentage maintained at about 60–70% (Table 2, Figure 1).

**Figure 2:**
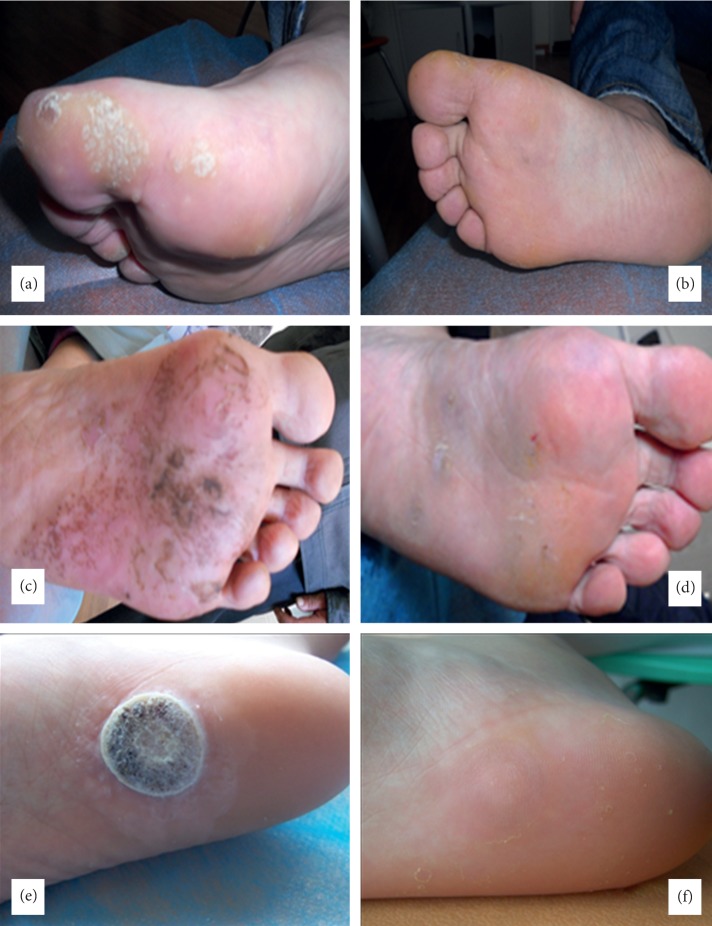
Three patients with verruca plantaris before (a, c, e) and after (b, d, f) the treatment.

Concerning of verruca plantaris, about 20% patients achieved at least 50% improvement after 1 session of treatment. The percentage sharply increased to more than 53% after 2 sessions of treatment and to more than 85% after 3 sessions of treatment. It reached a peak percentage of 90.60% after 4 sessions of treatment. After that, the percentage maintained at about 74–88% (Table 3, Figure 2).

**Figure 3:**
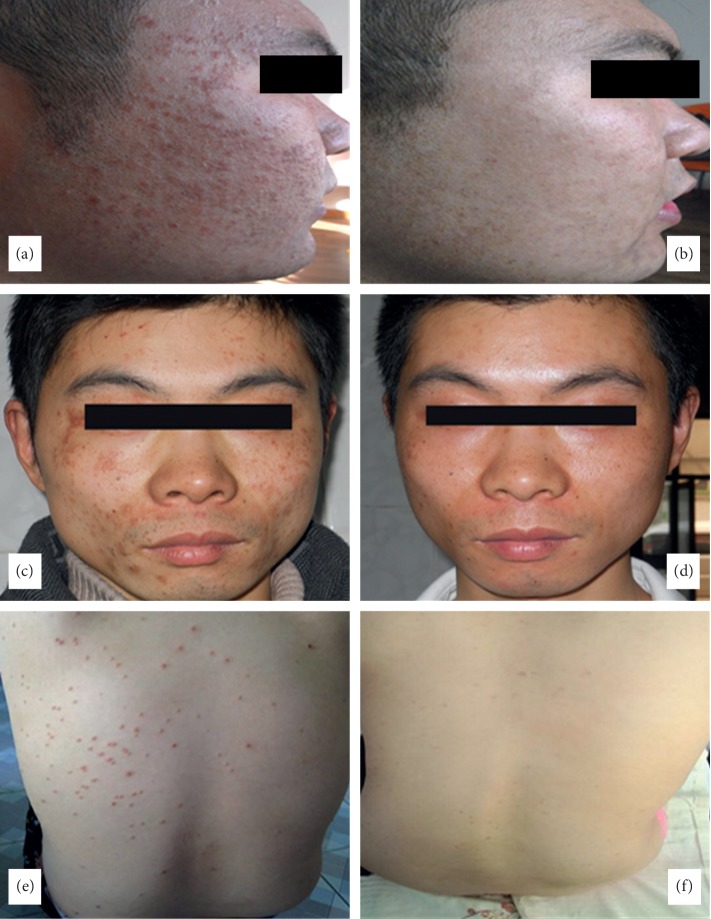
Three patients with verruca plana before (a, c, e) and after (b, d, f) the treatment.

And for verruca plana, about 43% patients achieved at least 50% improvement after 1 session of treatment and maintained at this level after 2 sessions. The percentage rapidly went up to more than 69% after 3 sessions of treatment. It reached a peak percentage of 80.91% after 4 sessions of treatment. After that, the percentage maintained at about 62–77% (Table 4, Figure 3).

### 3.3. The Results of 4 Sessions of Treatment

**Figure 4:**
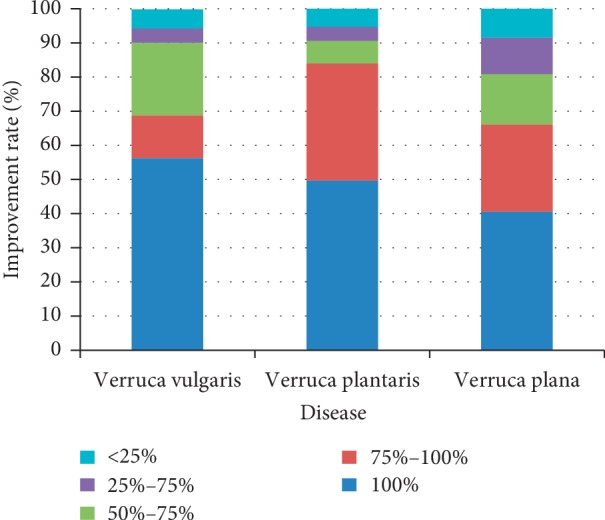
The percentages of treatment responses in the three diseases after 4 treatment sessions.

The percentage of patients who achieved at least 50% improvement was 90.34% for verruca vulgaris, 90.60% for verruca plantaris, and 80.91% for verruca plana. Differences were statistically significant among the three diseases (*χ*^2^ = 14.416, *p*=0.001). The efficacy of verruca vulgaris or verruca plantaris was better than that of verruca plana (*Z* = 3.012, *p*=0.003; *Z* = 3.580, *p*=0.000). The efficacies of verruca vulgaris and verruca plantaris were similar (*Z* = 0.029, *p* > 0.05).  Figure 4 shows the percentage of treatment responses in the three diseases after 4 sessions of treatment.

### 3.4. Adverse Effects and Recurrence

During the treatment, the most common adverse reactions were erythema, slight edema, crusting, and desquamation. After the warts fell off, isometrical wounds would form and patients would feel slight to moderate pain. All the adverse effects resolved soon, especially after using the sea buckthorn oil. No allergic or aggravated signs, including itching, severe erythema, severe edema, or exudation, developed in any patients. At month 3, respective 28 verruca vulgaris, 28 verruca plantaris, and 18 verruca plana patients received telephone follow-up, and no recurrence was reported.

## 4. Discussion

Topical agents such as salicylic acid and cantharidin may have beneficial effect on verruca vulgaris and verruca plantaris [21–23]. But these agents should not be used by patients themselves theoretically. Because it is difficult for patients to judge the depth of lesions and dermal condition, and the corrosive topical agents may increase the possibility of tissue damage [24]. Physical removal methods such as surgical excision and electrodessication may lead to scars and tend to have longer healing time [25]. Another commonly used method of physical destruction is cryotherapy. Despite not leaving scars, cryotherapy is not more effective than topical salicylic acid in some cases [21–23]. Besides, these aggressive treatments often cause intolerable pain, hyperpigmentation, hypopigmentation, or occasionally allergic contact dermatitis [26, 27]. These adverse effects may make both dermatologists and patients hesitate to start the treatment [8]. Currently, there is no standard method of treatment that is highly effective and without side effects. It is necessary to develop a reliable, easy-to-use, effective, economical minimal side-effect treatment for verruca.

The present study was a retrospective and multicenter study. The results were based on a relatively large-scale survey of over 1800 patients. Topical treatment with YIKEER enabled over 80% patients (regardless of verruca vulgaris, verruca plantaris, or verruca plana) to achieve complete response. Our overall efficacies were better than those of previous studies, such as treating with cryotherapy or neodymium-doped yttrium aluminium garnet laser with success rates of 63–70% [2, 28]. Specifically, the percentage of patients with verruca vulgaris or verruca plantaris who achieved complete response was over 86% by using the YIKEER, and the percentage of verruca vulgaris patients who achieved complete response was higher than 65% by using topical viable bacillus Calmette–Guerin, and similarly, the percentage of verruca plantaris patients who achieved complete response was higher than 79.1% by a single sublesional injection of interferon-α2a [4, 29]. In addition, 82.42% verruca plana patients gained the complete response by using YIKEER, which was higher than 53.33% auricular acupuncture in a previous study [8].

At least four treatment sessions of YIKEER were needed to gain complete or partial responses in most patients. The percentage of patients who achieved at least 50% improvement was about 90% for verruca vulgaris or verruca plantaris, which was higher than the 80.91% for verruca plana. It indicated that the efficacy of verruca vulgaris or verruca plantaris was better than that of verruca plana. However, the onset time of verruca vulgaris or verruca plantaris seemed to be relatively slower than that of verruca plana. As after 1 session of treatment, the percentages of patients with former two verrucae who achieved at least 50% improvement were about 7% and 20%, in contrast to about 43% of verruca plana patients. After that, the efficacy of verruca vulgaris or verruca plantaris increased markedly and the efficacy of verruca plana became gentle.

In the present study, no adverse effects, such as hyperpigmentation or scarring, and no allergic or aggravated signs were observed. Although the treatment duration was longer than cryotherapy or laser, it was easier to be accepted by patients for the minimal pain. The patients who received telephone follow-up at 3 months all reported no recurrence, implying a low recurrence rate by the treatment.

Some limitations existed in the current study. Firstly, there were a small number of patients who showed no response to the treatment. The reasons may be as followings: patients being not sensitive to drugs, body resistance being not strong enough, and reexposure to infection. Secondly, the treatment methods did not unify to an extent and varied with patients owing to individual difference. Thirdly, as the natural shortcoming of a retrospective study, no control group was set and thus the evidence level was not very high. Finally, although the 3 diseases are caused by the same virus, different virus subtypes and different clinical characters lead to diverse responses to the treatment.

## 5. Conclusion

To sum up, YIKEER is an effective, safe, easy-to-use, and well-tolerated agent for verruca including verruca vulgaris, verruca plantaris, and verruca plana. And it is worthy of further clinical trial to verify the current results.

## Figures and Tables

**Table 1:** Demographic characteristics of the patients with verruca vulgaris, verruca plantaris, and verruca plana.

	Verruca vulgaris	Verruca plantaris	Verruca plana
No. of patients	614	716	530
Gender			
Male (*n*)	318	340	168
Female (*n*)	296	376	362
Mean age (range)	36 (3–69) years	33 (4–67) years	29 (6–55) years
Treatment duration			
1 session (*n*)	0	0	0
2 sessions (*n*)	8	8	0
3 sessions (*n*)	40	42	30
4 sessions (*n*)	306	316	196
5 sessions (*n*)	56	152	102
6 sessions (*n*)	38	14	36
7 sessions (*n*)	40	26	30
8 sessions (*n*)	28	14	12
9 sessions (*n*)	70	116	106

**Table 2:** Lesion therapeutic response over the sessions of treatments on verruca vulgaris.

	Complete response	Partial response			No response
	100%	75%–100%	50%–75%	25%–50%	<25%
Session 1 (*N* = 586)	0	6	36	510	34
Session 2 (*N* = 586)	6	36	264	248	32
Session 3 (*N* = 578)	36	298	54	158	32
Session 4 (*N* = 538)	202	66	118	22	30
Session 5 (*N* = 232)	58	34	86	24	30
Session 6 (*N* = 176)	30	44	60	14	30
Session 7 (*N* = 138)	20	74	16	0	28
Session 8 (*N* = 98)	24	46	0	0	28
Session 9 (*N* = 70)	0	42	0	0	28

**Table 3:** Lesion therapeutic response over the sessions of treatments on verruca plantaris.

	Complete response	Partial response			No response
	100%	75%–100%	50%–75%	25%–50%	<25%
Session 1 (*N* = 686)	0	14	124	512	38
Session 2 (*N* = 686)	6	42	314	288	36
Session 3 (*N* = 680)	34	344	200	68	34
Session 4 (*N* = 638)	316	220	42	26	34
Session 5 (*N* = 322)	138	116	30	6	32
Session 6 (*N* = 170)	14	110	16	0	30
Session 7 (*N* = 156)	20	88	16	0	30
Session 8 (*N* = 130)	14	72	14	0	30
Session 9 (*N* = 116)	22	48	16	0	30

**Table 4:** Lesion therapeutic response over the sessions of treatments on verruca plana.

	Complete response	Partial response			No response
	100%	75%–100%	50%–75%	25%–50%	<25%
Session 1 (*N* = 512)	0	36	186	248	42
Session 2 (*N* = 512)	0	52	200	218	42
Session 3 (*N* = 512)	6	206	142	116	42
Session 4 (*N* = 482)	194	124	72	52	40
Session 5 (*N* = 286)	62	78	82	24	40
Session 6 (*N* = 184)	36	72	16	20	40
Session 7 (*N* = 148)	20	52	20	16	40
Session 8 (*N* = 118)	10	42	28	0	38
Session 9 (*N* = 106)	16	26	26	0	38

## Data Availability

The data used to support the findings of this study are available from the corresponding author upon request.

## Additional Points

*Key Points.* YIKEER is a kind of compound preparation of Chinese traditional medicine and has been used in the treatment of verruca for several years. The vast majority of patients were cured after using YIKEER. During the treatment, the most common adverse reactions were erythema, slight edema, crusting, and desquamation.
